# Do care homes deliver person-centred care? A cross-sectional survey of staff-reported abusive and positive behaviours towards residents from the MARQUE (Managing Agitation and Raising Quality of Life) English national care home survey

**DOI:** 10.1371/journal.pone.0193399

**Published:** 2018-03-21

**Authors:** Claudia Cooper, Louise Marston, Julie Barber, Deborah Livingston, Penny Rapaport, Paul Higgs, Gill Livingston

**Affiliations:** 1 UCL Department of Old Age Psychiatry, Division of Psychiatry, London, United Kingdom; 2 Camden and Islington NHS Foundation trust, London, United Kingdom; 3 Department of Primary Care and Population Health, UCL, London, United Kingdom; 4 PRIMENT Clinical Trials Unit, UCL, London, United Kingdom; 5 UCL Department of Statistical Science, London, United Kingdom; University of Exeter, UNITED KINGDOM

## Abstract

**Background:**

There are widespread concerns about abuse of care home residents. We report, in the largest care home survey, prevalence of staff anonymously-reported, perpetrated/witnessed abusive behaviours towards care home residents over 3 months. We also report positive care behaviours.

**Methods:**

1544 staff in 92 English care home units completed the revised Modified Conflict Tactics Scale and Maslach Burnout Inventory.

**Outcomes:**

Most staff reported positive care behaviours, but specific person-centred activities were sometimes infrequent. Many care home staff were never or almost never aware of a resident being taken out of the home for their enjoyment (34%, n = 520); or an activity planned around a resident’s interests (15%, n = 234). 763 (51%; 95% Confidence Interval (CI) 47% to 54%) of care home staff reported carrying out or observing potentially abusive or neglectful behaviours at least sometimes in the preceding 3 months; some abuse was reported as happening “at least sometimes” in 91/92 care homes. Neglect was most frequently reported: making a resident wait for care (n = 399, 26%), avoiding a resident with challenging behaviour (n = 391, 25%), giving residents insufficient time for food (n = 297, 19%), and taking insufficient care when moving residents (n = 169, 11%). 1.1% of staff reported physical and 5% verbal abuse. More staff reported abusive/neglectful behaviour in homes with higher staff burnout-depersonalisation scores (adjusted odds ratio 1.191, CI 1.052–1.349).

**Interpretation:**

Staff anonymous reports of abusive behaviour and neglect could be used to monitor care quality, as cases currently reported are probably tip of the iceberg, and be an outcome in intervention studies.

## Introduction

By 2021, an estimated million United Kingdom (UK) people will have dementia, despite a recent fall in incidence [1]. One third of UK people with dementia live in care homes and at least two thirds of care home residents have dementia [2]. A number of inquiries regarding abuse and neglect of residents have influenced public perceptions of care homes [3]. The UK Department of Health defines abuse as “a violation of an individual's human and civil rights by another person(s)" (2). Abuse is defined by the impact, rather than intention of actions or inactions on an individual. A carer may unintentionally be neglectful if they are unaware of a care recipient’s needs.

Conceptual models developed to try to explain elder abuse consider victim vulnerability, abuser stress, psychopathology or impairment, intra-individual dynamics and societal attitudes [4]. Most care home residents have dementia, rely on others for personal care and many exhibit challenging behaviours, factors associated with higher risk of abuse [5, 6]. Carer stress is associated with: low job satisfaction, long hours, low pay, physical demands, staff shortages and minimal education and training [4, 7] and lower empathy which may be linked to lower care quality [8]. In family carers, experiencing violence and aggression from people with dementia was a significant predictor of acting abusively, possibly because staff react defensively or find managing aggression stressful. This may also be true for paid carers and could explain why people with dementia who have more neuropsychiatric symptoms are at increased risk of abuse [9].

In previous surveys, a quarter of relatives of care home residents reported incident(s) of physical abuse [10]; 16% of 114 long term care staff reported committing significant psychological abuse [11]; and over 80% of 577 nursing home staff observed abuse although far fewer admitted to acting abusively [12]. As care workers reporting abuse face potential adverse legal, employment and social consequences, recent surveys have elicited care worker experiences anonymously. In an Israeli study of 510 care staff completing an anonymous questionnaire, just over half admitted abuse and 70% had witnessed maltreatment in the past year; more abuse was reported by staff who experienced more burnout and worked in larger facilities with higher staff turnover [13]. In a small UK survey in 5 nursing homes, most respondents (n = 138, 88.5%) reported witnessing or suspecting abuse in homes where they had previously worked [14]. In both these surveys, staff were asked to identify incidence of “abuse” or “maltreatment”, so behaviours not identified correctly as abusive were undetected. There have been no previous anonymous surveys of elder abuse to elicit behaviours such as ignoring residents’ needs or making them wait for care, without requiring staff to identify behaviours as abusive. As many professionals do not correctly identify abusive behaviours, this is an important omission [15].

The MARQUE (Managing Agitation and Raising Quality of Life) cohort study, primarily devised to consider the relationship between agitation, carer coping and quality of life, enabled us to examine care home abuse on a large scale. We included our recently developed measure for anonymous reporting of abuse and neglect in care homes in baseline data collection and used this to describe the prevalence of abuse and its relationship with care home and staff characteristics. We based our hypothesis on the conceptual model of abuse outlined above, and in particular the likelihood that abuse and neglect occurred most often where there was greater resident vulnerability, abuser stress strained intra-individual dynamics. We hypothesised that staff were more likely to report abusive behaviour in care homes:

1. where staff were more stressed, as indicated by higher mean staff burnout depersonalisation scores;
2. with more residents and
3. fewer permanent staff, because staff-resident dynamics were more challenged;
4. where residents with dementia were potentially more at risk of abuse because they had higher mean neuropsychiatric inventory scores; and
5. in care homes with lower environment quality scores.

## Materials and methods

### Setting and sampling

Harrow Research Ethics Committee (14/LO/0034) gave permission for the study. We recruited care homes from across England, from July 2014 to October 2015, of each provider type (voluntary, state and private), care provision (nursing, residential) and urban/suburban and rural locations. Sample size was determined by the MARQUE cohort study primary objective which was to examine the longitudinal relationship between carer coping and patient quality of life and its dependence on severity of agitation. The study aimed to recruit 2537 residents from 72 care home units (35 residents per care home) [16].

### Procedures

We recruited care homes through third sector partners, NHS (National Health Service) trusts, Department of Health publicity and the NIHR (National Institute for Health Research) Clinical Research Network. We sought care home managers’ agreement for their care home’s inclusion. In included homes, all consenting regular care team members (whether permanent or regular bank staff), who provided hands-on care were asked to complete measures. We excluded agency staff as we sought to recruit care workers who regularly worked in the homes. We identified residents with a dementia diagnosis and for others completed the Noticeable Problems Checklist [17] with staff to detect residents with undiagnosed probable dementia. We asked the paid carer working most closely with each resident with dementia to complete proxy measures (measures completed by the staff member reporting their understanding of the wellbeing and illness experienced by the resident). All participants gave informed, written consent before taking part.

### Measures

Trained research assistants conducted assessments at the care homes. Data was collected in the baseline MARQUE study interview [16].

#### Care home measures

We recorded characteristics of the included care homes (see Table 1 for details recorded); where care homes comprised units each staffed by discrete staffing groups, we recorded these as separate care home units. The physical environment of care homes was assessed by the TESS (Therapeutic Environment Screening Survey for Nursing Homes and Residential Care), an instrument with good inter-rater reliability and concurrent validity [18]. The TESS score is the sum of 15 items, each scored 0–2 (facility maintenance, cleanliness, handrails, call buttons, light intensity, light glare, light evenness, hallway length (shorter is better), homelikeness, room autonomy, the presence of telephones, tactile stimulation, visual stimulation, privacy, and outdoor areas). Higher scores indicate better environmental quality [19].

**Table 1:** Characteristics of care home studied.

		n (unless stated)	% (unless stated)
**Care home (CH) level (N = 92 unless stated)**			
Home type	Nursing	12	13
	Personal care	39	42
	Nursing and personal care	41	45
Home Manage-ment	Private	74	80
	Council	4	4
	Charity	12	13
	Other	2	2
Dementia specialism	Dementia registered	81	88
	Dementia specialist	41	45
Permanent staff in the last 7 days (at work and on leave) **median (IQR)** N = 91		30	(20, 49)
Agency/ bank staff in the last 7 days **median (IQR)** N = 90		0	(0, 1)
Number of residents **median (IQR)** N = 91		34	(24, 50)
Number of residents with dementia **median (IQR)** N = 91		26	(17, 38)
Staff: resident ratio **mean (sd)** N = 91		0.99	(0.44)
Environmental quality score on the TESS N = 82		16	(3)
**Mean (sd)** staff burnout (per CH)	emotional exhaustion N = 89	15	(4)
	personal accomplishment N = 86	39	(2)
	Depersonalisation N = 86	3	(1)
**Mean (sd)** NPI (per CH) N = 92		14	(8)
**Mean (sd)** staff proxy DEMQOL (per CH) N = 92		100	(6)
**Mean (sd)** percentage with dementia severity severe or very severe from CDR (per CH) N = 92		70	(22)
**Staff level (those that completed the abuse questionnaires) (N = 1544)**			
Home type	Nursing	188	12
	Personal care	609	39
	Nursing and personal care	747	48
Home manage-ment	Private	1227	79
	Council	91	6
	Charity	181	12
	Other	45	3
Dementia specialism	Dementia registered	1412	91
	Dementia specialist	704	56

#### Carer measures

Staff completing measures for the MARQUE baseline survey were also asked to complete the revised Modified Conflict Tactics Scale for professional carers (primary outcome), a measure of helpful and potentially abusive behaviour perpetrated or witnessed by staff which we have developed and piloted with care home staff and found to be acceptable and have content validity [20]. It is based on a previous measure used extensively among people with dementia and family carers [6, 21, 22]. It comprises 10 potentially abusive items and six positive care items (Tables 2 and 3. Care staff were asked whether each item had, in the last three months happened “never”, “almost never”, “sometimes” “most of the time” or “all of the time”. The carers self-completed this questionnaire anonymously, in private and sealed it in a blank envelope. We recorded which care home the person worked in. We could not identify individual participants, but notified the care home manager where staff reported that residents had been hit or shaken. If residents or staff told us or we witnessed potentially abusive behaviour this was handled according to our protocol in line with UK safeguarding procedures.

**Table 2:** Proportion of care staff reporting abusive behaviour at least sometimes by care home type.

Variable	Reporting abuse happens at least sometimes	
	n/N	%
**Ownership**		
Private	614/1202	51
Council	43/89	48
Charity	92/177	52
Other	14/43	33
		P-value* = 0.110
Private	614/1202	51
Other	149/309	48
		P-value* = 0.370
**Type of care home**		
Nursing	96/185	52
Personal care	288/593	49
Nursing and personal care	379/733	52
		P-value* = 0.483
Dementia registered	704/1382	51
Not dementia registered	59/129	46
		P-value* = 0.258
Dementia specialist	339/685	49
Not dementia specialist	424/826	51
		P-value* = 0.476

* P-values from Chi squared test

**Table 3:** Number (%) of care home staff reporting that they had seen or carried out each of the potentially abusive behaviours studied at least sometimes in the last 3 months.

		N(%) giving each response					Endorsing sometimes or more frequent occurrence N(%)	
	N	Never	Almost never	Some-times	Most of the time	All of the time	Of all carers	Care homes with >= 1 carer reporting
**Physical and verbal abuse**								
Hit or shaken a resident	1537	1523 (99%)	13 (1%)	1 (0.1%)	-	-	1 (0.1%)	1 (1%)
Threatened to use physical force on a resident	1537	1481 (96%)	35 (2%)	12 (1%)	2 (0.1%)	7 (0.5%)	21 (1%)	19 (21%)
Shouted, insulted or spoken harshly to a resident	1539	1320 (86%)	145 (9%)	68 (4%)	3 (0.2%)	3 (0.2%)	74 (5%)	49 (53%)
**Neglect**								
Made a resident wait for care	1532	731 (48%)	402 (26%)	353 (23%)	26 (2%)	20 (1%)	399 (26%)	88 (96%)
Avoided a resident with challenging behaviour	1535	909 (59%)	235 (15%)	334 (22%)	31 (2%)	26 (2%)	391 (25%)	84 (91%)
Not given a resident enough time for food	1529	1000 (65%)	232 (15%)	254 (17%)	24 (2%)	19 (1%)	297 (19%)	81 (88%)
Not taken enough care when moving a resident	1526	1153 (76%)	204 (13%)	117 (8%)	19 (1%)	33 (2%)	169 (11%)	67 (73%)
Ignored a resident while giving care or when they ask for help	1534	1221 (80%)	194 (13%)	98 (6%)	3 (0.2%)	18 (1%)	119 (8%)	58 (63%)
Isolated a resident	1531	1389 (91%)	73 (5%)	56 (4%)	9 (1%)	4 (0.3%)	69 (5%)	45 (49%)
Told a resident they will be sent away	1534	1466 (96%)	37 (2%)	24 (2%)	4 (0.3%)	3 (0.2%)	31 (2%)	22 (24%)
*Any abusive behaviour (at least sometimes)*	*1511*						*763 (51%; 95% CI 47%*, 54%*)	*91 (99%; 95% CI 94%*, *100%)*

*95% CI (Confidence Interval) calculated based on the clustered sandwich estimator to account for clustering by care home.

The following measures were completed as part of the MARQUE baseline assessment; since they could not be directly linked to anonymous abuse reports, they were analysed as mean values at the care home unit level:

The *Maslach Burnout Inventory* (MBI): a commonly used measure of burnout in care home staff, with adequate psychometric properties [23]. Burnout describes physical, mental and emotional exhaustion that may be accompanied by a change in attitude, from positive and caring to negative and unconcerned. The MBI comprises three subscales—asking how often certain feelings or behaviours associated with emotional exhaustion, depersonalisation and personal accomplishment are currently happening, on a 7 item scale, from never to every day. It does not specify a time period, as burnout is conceptualised as an enduring state [24]. We hypothesised prior to analysis that the depersonalisation (unfeeling and impersonal response towards care recipients) subscale would be associated with abusive behaviour.

The paid carer working most closely with each resident with dementia completed the following measures:

- The Dementia Quality of Life (DEMQOL) proxy, a responsive, valid and reliable measure of quality of life in people with dementia in the last week. It is a 31-item interviewer-administered questionnaire [25].
- Staff gave information so the researcher could rate dementia severity using the Clinical Dementia Rating (CDR), a reliable and valid instrument for rating performance in Memory, Orientation, Judgment and Problem solving, Community Affairs, Home and Hobbies, and Personal Care. This information was used to classify residents’ dementia severity into: very mild, mild, moderate or severe [26].
- The Neuropsychiatric Inventory (NPI), a validated instrument was used to evaluate neuropsychiatric symptoms in the past 4 weeks. 12 domains, each rating a discrete symptom are scored between 0 and 12 (calculated as the product of symptom’s frequency (0–4) and severity (0–3)) with higher scores meaning increasing severity. A summed scored can be calculated for total neuropsychiatric symptoms [27].

### Analysis

We analysed data using Stata version 14. We determined the prevalence of reporting that each individual behaviour was happening “at least sometimes”; and of reporting each positive behaviour was happening “never” or “almost never”. We reported these as prevalence amongst care homes and carers. The overall prevalence of any abusive behaviour reported at least sometimes was also calculated with a 95% confidence interval. When estimating prevalence by carer we used a sandwich estimate of the standard error to account for clustering [28].

Mixed effects logistic regression models were used to examine our hypotheses that staff in care home units with (a) more residents (b) lower staff: resident ratios (c) higher mean MBI depersonalisation subscale scores (d) lower TESS scores; and (e) higher mean NPI scores for residents; were more likely to report that any abusive behaviour was happening at least sometimes. These factors were all included as fixed effects in the models while clustering by care home unit was accounted for as a random effect. We fitted unadjusted models and models adjusted for the number of staff working in care home units (a fixed effect), because in home units with more staff, the likelihood of witnessing a staff member acting abusively would be logically greater.

## Results

### Description of survey

Of the 97 care home units participating in the MARQUE study baseline interview, 92 participated in this survey. Five care homes were not invited to participate as we had not yet gained ethical approval for this study component when they took part in MARQUE; one of these homes provided nursing care, the others nursing and personal care; all were dementia-registered, one was a dementia-specialist home. The five excluded homes had more permanent staff (median (Interquartile Range, IQR) 68 (49,80) versus 30 (20, 49)), and more residents (53 (51, 85) versus 34 (24,50)) than those that participated.

Of the 2120 care home staff approached, 1702 (80.3%) took part in the MARQUE study. Of the 1702 care home staff participating in MARQUE 1544 (91%) took part in the abuse study. Of the 158 participating in MARQUE but not the abuse study, 138 worked in the five homes not invited to take part and 20 declined participation.

Table 1 describes characteristics of the included care homes. In these care homes, 1341 (86%) of MARQUE study respondents were female, 1080 (30%) spoke English as a second language; 83 (5.4%) had no qualifications; 539 (35%) were educated to GCSE (General Certificate of Secondary Education)/NVQ (National Vocational Qualification) Level 2; 500 (32%) to A-Level/NVQ Level 3–5; 318 (21%) to degree or post-degree level. Among those participating, 193 (12%) had a nursing qualification. As only 20 staff MARQUE participants declined to participate in this study, these characteristics approximate those of the anonymous participants in this study.

### Potentially abusive behaviours

Carrying out or observing one or more potentially abusive or neglectful behaviour at least sometimes in the preceding 3 months was reported by 763 (51%; 95% Confidence Interval (CI) 47% to 54%) of care home staff. Some abuse was reported as happening “at least sometimes” in 91/92 care homes. The proportion of staff reporting abuse was similar across care home types (Table 2). Only 14 (1.1%) of care home staff reported that residents had been hit or shaken, and 56 (4%) were aware of threats of physical violence to residents (Table 3). Staff being verbally abusive to residents (shouting at, insulting or speaking harshly to them) at least some of the time was reported by 74 (5%) of respondents. The most frequently reported behaviours were neglectful: making a resident wait for care (n = 399, 26%), avoiding a resident with challenging behaviour (n = 391, 25%), not giving enough time for food (n = 297, 19%), and not taking enough care when moving a resident (n = 169, 11%).

### Positive behaviours

The majority of care staff reported that most of the time staff spoke nicely to residents during personal care (98%, n = 1490), enjoyed spending time keeping them company (57%, n = 886) and spent time to get to know them (63%, n = 966). By contrast, 520 (34%) of care home staff were never or almost never aware of resident being taken out of the home for their enjoyment; 196 (13%) had never or almost never experienced relatives being involved in care planning, and 234 (15%) had never or almost never been aware of an activity planned around a resident’s interests (Table 4).

**Table 4:** Number (%) of care home staff reporting that they had never or almost never seen or carried out each of the positive behaviours studied in the last 3 months.

		N(%) giving each response					Endorsing never or almost never occurrence	
	N	Never	Almost never	Sometimes	Most of the time	All of the time	Of all carers	Care homes with >= 1 carer reporting
Taken resident out for their enjoyment	1516	412 (27%)	108 (7%)	633 (42%)	218 (14%)	145 (10%)	520 (34%)	89 (97%)
Planned an activity that fits with their interests	1518	172 (11%)	62 (4%)	419 (28%)	505 (33%)	360 (24%)	234 (15%)	78 (85%)
Involved a resident’s family in care planning	1483	167 (11%)	29 (2%)	233 (16%)	308 (21%)	746 (50%)	196 (13%)	78 (85%)
Spent time getting to know a resident	1534	13 (1%)	14 (1%)	121 (8%)	447 (29%)	939 (61%)	27 (2%)	23 (25%)
Enjoyed spending time with a resident just to keep them company	1539	14 (1%)	8 (0.5%)	215 (14%)	438 (28%)	864 (56%)	22 (1%)	21 (23%)
Talked to a resident nicely while giving personal care	1527	18 (1%)	3 (0.2%)	16 (1%)	227 (15%)	1263 (83%)	21 (1%)	18 (20%)

### Associations with potentially abusive behaviour

A greater odds of staff reporting that any potentially abusive behaviour happened at least sometimes was associated with working in a home with higher staff burnout depersonalisation scores (1.19, 95% CI 1.05–1.35). Contrary to our hypothesis, numbers or ratios of staff to residents, environmental quality and neuropsychiatric symptom severity in residents with dementia were not associated with abuse. There was a small association with staff reporting higher resident quality of life (1.03, 95% CI 1.01–1.06) (Table 5).

**Table 5:** Adjusted and unadjusted associations of proportion of staff reporting abusive behaviours* occur at least sometimes and other factors studied (p<0.05 in bold).

	Unadjusted N = 1511 max		Adjusted N = 1484 max*	
Variable	Odds ratio**	95% CI	Odds ratio**	95% CI
Number of residents	1.007	(1.000, 1.014)	1.010	(0.999, 1.021)
Staff: resident ratio	0.870	(0.638, 1.188)	†	
Number of permanent staff	1.003	(0.997, 1.009)		
Number of agency/ bank staff	1.006	(0.921, 1.098)	1.002	(0.917, 1.095)
**Staff burnout—depersonalisation**	**1.186**	**(1.047, 1.343)**	**1.191**	**(1.052, 1.349)**
Environmental quality score on the TESS	0.964	(0.920, 1.010)	0.959	(0.916, 1.004)
NPI	0.986	(0.971, 1.002)	0.985	(0.969, 1.001)
**Mean staff proxy DEMQOL**	**1.033**	**(1.010, 1.056)**	**1.031**	**(1.008, 1.055)**
Mean percentage with CDR severe or very severe dementia	1.001	(0.995, 1.007)	1.000	(0.994, 1.006)

* Adjusted for number of permanent staff

^†^ Adjustment not done as staff: resident ratio includes number of permanent staff.

** per unit increase in variable indicated

CI Confidence Interval

## Discussion

This is the first large survey of abuse and neglect in English care homes, and the largest survey to date of abuse and neglect in care homes in any country. Just over half of respondents reported that potentially abusive or neglectful behaviour towards residents occurred at least sometimes. Some abuse or neglect was reported in all but one of the 92 care home units. Neglectful behaviours were most common, and very few care home workers reported actual or threatened physical abuse. While only 5% of carers interviewed reported verbal abuse, at least one carer endorsed this item in over half of the homes. As there may be under-reporting, due to fear of reprisal or lack of awareness of abuse or neglect occurring, these are likely minimum estimates of the prevalence of abuse and neglect. Our worrying findings accord with previous studies [13]. Under section 42 of the Care Act (2014), safeguarding adults is a statutory duty in England. In 2015–16, 39,485 cases of vulnerable adult care home residents (of all ages) experiencing, or being at risk of abuse and neglect were investigated and concluded. Most reported cases related to neglect (34%), with physical abuse next most common (27%) [29]. As nearly 15,000 care homes in England are registered with the Care Quality Commission (CQC) [30], our findings suggest that the neglect investigations currently initiated in care homes may be the tip of the iceberg [31]. Physical abuse was rarely reported in our survey, but constitutes a significant proportion of reported concerns, suggesting cases are more likely to be reported to statutory services.

While the concept of person-centred care [32] is widely accepted by researchers and care home managers, there appears to be a gap in its implementation in care homes. Staff are often caring for residents who resist care, or are verbally or physically aggressive, with little training or support. Where emotional distancing and objectifying behaviours are used as ways to cope with this, there is clear dissonance with the concepts of person-centred care. Attributing aggression to dementia rather than the person may, for example, help staff to be less distressed by it, but perhaps normal inter-personal interactions become more difficult in circumstances where some behaviours are viewed at two different levels; those of the person and those of the dementia [33].

We explored putative associates of abusive behaviour at the care home level, based on our selected conceptual model of abuse occurrence as related to staff stress and distress, resident vulnerability and intra-individual dynamics [4]. Abuse or neglect, as hypothesised, was reported more in homes where staff experienced more burnout and depersonalisation feelings towards residents, mirroring findings with dementia family carers [34]. More abuse was reported in units where staff rated the quality of life of residents with dementia higher. This might be a chance association, or perhaps indicative that staff experiencing more burnout were more likely to act abusively or neglectfully and empathise or notice less when residents were distressed. Staff may conflate life quality of residents with care quality. Perhaps those experiencing more burnout worked to a less person-centred, more task focussed model, rating residents’ quality of life more highly when care tasks were completed to schedule.

Our hypotheses that abuse happened more in care home units where residents with dementia had more neuropsychiatric symptoms that increased their vulnerability, or with fewer permanent staff per resident and more residents because staff had less time to deliver person-centred care, were unsupported. Perhaps this was because most homes operated at minimum legal staffing levels, with additional staff employed only when highly agitated residents required 1:1 care. Staff numbers tend to be higher in care homes where more residents have clinically significant agitation [16]. Having more staff per resident in a unit probably indicates that residents need more care, not that staff work under less pressure, with more time to provide person-centred care. Our hypothesis that environmental quality was associated with less abuse was also unsupported. While important for many residents and for staff, a more pleasant environment was insufficient to reduce likelihood of abuse.

Positive care behaviours greatly outnumbered abusive or neglectful behaviours. Carers reported that most of the time, staff spoke nicely to residents, and spent quality time, trying to know them better. However, activities that took more planning and time—involving families in care planning, and planning activities around a resident’s interests—were less frequent. A third of care workers reported that residents were rarely taken out of the home for their enjoyment. Perhaps staff lacked the time to plan activities, or this was not perceived as within their role or power. Taking residents out may have been avoided due to the possibility of falling, or precluded by staffing levels at minimum levels. Absence of pleasant events and activities may in itself be neglect—albeit unintentional neglect. Reducing abuse and neglect in care homes, requires, in our view, a systemic approach, where they are not perceived as the commission or omission of a direct carer, but as occurring in a system. In its widest view, this involves a society that has delegated care of the most vulnerable to carers undertaking challenging, stressful work for low pay with minimum training and support.

Our study demonstrates that anonymous reporting of abuse by care home workers is acceptable and feasible, and it could be a new, useful indicator of care quality for home managers, regulatory bodies and researchers. The only existing evidence-based interventions to reduce elder abuse target use of physical restraint, and were developed in countries where restraint is acceptable in some circumstances [35]. There are no interventions known to effectively reduce less tangible abusive behaviours, partially due to difficulties measuring outcomes that paid carers are unwilling and residents with dementia unable or unwilling to report.

Our study suggests that that future abuse interventions should focus on reducing staff burnout and depersonalisation; introducing true person-centred care by encouraging staff to explore residents’ personal histories, current and past interests and build pleasant interactions into care, reducing objectification of residents would from our findings, be rational strategies. We are currently evaluating such an approach. We found no association between care home size, staffing numbers and abuse likelihood, perhaps because the care homes included were similarly staffed, or because level of staff training and support, which we did not measure, matters, rather than numbers of staff alone.

Our measure of abuse and neglect was developed with care home workers; it is valid, and the use of anonymous reporting reduced potential for desirability bias. We did not require staff to identify behaviours as maltreatment, so we could capture potentially abusive behaviour that observers or perpetrators may not have identified as abusive. We cannot estimate the proportion of staff acting abusively from our findings, because we did not ask staff to differentiate witnessed from committed abuse [36]. Our work with care staff developing the measure suggested this would have reduced its acceptability. The measure is intended as an indicator of abuse prevalence within a home rather than a way of directly identifying abusing staff. Previous surveys that have distinguished between observed and perpetrated abuse found that observed abuse was reported far more frequently, either because staff were unwilling to admit acting abusively or because abuse was perpetrated by few but seen by many [37]. In a more recent study, many staff were willing to report perpetrating abuse, so we will consider introducing this distinction in future studies [13].

Our questionnaire did not capture behaviour intent or context. We did not recruit a random probability sample of care homes, and those homes participating may have been more highly functioning, with more open cultures and proactive managers and thus with lower abuse rates than those declining participation. This may have biased our findings and potentially underestimated overall levels of abuse. We included regular bank workers but excluded agency staff who did work in the homes on a regular basis. We told staff that we would advise care home managers if concerning abuse were reported, although individual reporters were anonymous. This may have deterred some reporting due to fear of investigations, dismissal or blame. While abuse is always of concern, we told the home when reported abuse crossed a ‘red line’. We informed managers there was “concerning abuse” if any physical abuse was reported at all, or if any of the abusive items were reported as happening “most” or “all of the time”. Our results indicate that the proportion of carers reporting this level of abuse was low. We analysed associations between mean scores and abusive behaviour reports at care home unit level, as abuse was reported anonymously; power for these analyses was therefore limited by the care home unit sample size.

## Conclusions

Clinicians treating care home residents should be aware that neglect is common in care homes and that person-centred activities such as trips out or activities tailored to residents’ interests are often happening very infrequently; as these are possible targets for psychological interventions to improve wellbeing. Measuring harm to vulnerable people anonymously brings ethical dilemmas, as abuse detected cannot be directly addressed. Not measuring it, so it remains invisible, is a greater harm.
